# Letter to the Editor: “Analysis of the Interaction of Dp44mT with Human Serum Albumin and Calf Thymus DNA Using Molecular Docking and Spectroscopic Techniques”

**DOI:** 10.3390/ijms17111916

**Published:** 2016-11-16

**Authors:** Angelica M. Merlot, Sumit Sahni, Darius J. R. Lane, Vera Richardson, Michael L. H. Huang, Danuta S. Kalinowski, Des R. Richardson

**Affiliations:** Department of Pathology and Bosch Institute, University of Sydney, Sydney 2006, Australia; amer7408@uni.sydney.edu.au (A.M.M.); sumit.sahni@sydney.edu.au (S.S.); darius.lane@sydney.edu.au (D.J.R.L.); vera.richardson@sydney.edu.au (V.R.); michael.huang@sydney.edu.au (M.L.H.H.); danuta.kalinowski@sydney.edu.au (D.S.K.)

**To the Editor:**

In reading the article by Xu, Z., et al. entitled “Analysis of the Interaction of Dp44mT with Human Serum Albumin and Calf Thymus DNA Using Molecular Docking and Spectroscopic Techniques” [1], there is evidence that some of the key methods and comparisons utilized in this study are non-optimal and problematic.

We would also like to bring to the attention of readers a number of important flaws and factually incorrect statements in this article.

The paper examines the binding of the anti-cancer agent, di-2-pyridylketone 4,4-dimethyl-3-thiosemicarbazone (Dp44mT), to human serum albumin (HSA) and DNA and states (page 1, Introduction para 1) that “*no studies have examined the effects of the interaction between Dp44mT and biological molecules, such as proteins and nucleic acids*”. However, two studies from our group have previously comprehensively examined the binding of Dp44mT to HSA [2] and DNA [3]. These previous studies performed very similar experiments, including cytotoxicity studies, circular dichroism (CD), fluorescence and molecular docking studies, and hence, the current paper appears as a duplication and cannot be considered a novel contribution to the literature. Significantly, the authors knew of one of these articles as they directly cite it (REF #13 in their manuscript). Such statements appear misleading and inappropriate and this requires correction.

Moreover, the following methods, comparisons and interpretations of results may be unreliable and should be taken into consideration when interpreting the results presented:

- **Characterization and purity of Dp44mT:** The authors synthesized Dp44mT in their own laboratory and present only the mass spectrum to confirm its structural identity. Notably, other methods of structural characterization (e.g., ^1^H- and ^13^C-NMR, etc.) and chemical purity (e.g., elemental analysis) are required to ensure that the Dp44mT was indeed correctly synthesized, and was of the high purity necessary for biological studies. As such, the purity of the compound utilized is unclear.
- **The MTT-based proliferation studies:** The MTT studies performed in this article may be unreliable due to the fact that HSA (one of their key experimental variables) causes false positive results in MTT assays, producing spurious and inaccurate interpretations [4]. Alternative techniques should have been used, such as viable cell counts. Hence, the results obtained cannot be considered as reliable.
- **Comparison of HepG2 proliferation studies to previous literature:** The effect of HSA on the anti-proliferative activity of Dp44mT was performed using HepG2 cells in this article. Notably, the authors then compare the results obtained in this cell line to previous studies [2] and state (Page 2, last paragraph): “*growth inhibition induced by Dp44mT in the presence of equimolar HSA was similar to that without HSA, in contrast to previous findings [REF13: Merlot, A.M.; et al. Potentiating the cellular targeting and anti-tumor activity of Dp44mT via binding to human serum albumin: Two saturable mechanisms of Dp44mT uptake by cells. Oncotarget* ***2015****, 6, 10374–10398.].*” Significantly, the referenced study examined the effect of HSA and Dp44mT on the proliferation of SK-N-MC cells [2], *not* HepG2 cells, and thus, cannot be directly compared.
- **Circular dichroism method—use of DMSO**: DMSO has high far-UV absorbance and should not be used in CD measurements [5]. The implementation of DMSO leads to high tension (HT) voltage values and the saturation of the detector [5], leading to erroneous results. Alternative solvents that do not interfere with CD spectra, including ethanol and acetonitrile, should be used to dissolve the ligand for subsequent addition to the protein.
- **Circular dichroism interpretation:** Notably, upon examination of their experimental CD results, the authors state (Page 9, paragraph 1): “*In the presence of different concentrations of Dp44mT, a slight reduction in negative ellipticity was observed, indicating that the α-helix content of the protein was decreased (Figure S2). The contents of secondary structure can be calculated based on experimental data [REF36: Kandagal, P.B.; Ashoka, S.; Seetharamappa, J.; Shaikh, S.M.T.; Jadegoud, Y.; Ijare, O.B. Study of the interaction of an anticancer drug with human and bovine serum albumin: Spectroscopic approach. J. Pharm. Biomed. Anal.* ***2006****, 41, 393–399], but it was limited due to larger errors.”* However, the nature of the large error is not well explained. Unfortunately, the authors rely on simulated data to assess the effect of Dp44mT on the secondary structure of HSA. Critically, the simulated secondary structural data for HSA alone reported by the authors does not reflect the known levels of α-helical or β-sheet content of HSA previously published [6]. Hence, again, there are serious uncertainties regarding these data.

We hope this letter helps to resolve future interpretation and experimental problems of readers.
